# The minimal kinome of *Giardia lamblia *illuminates early kinase evolution and unique parasite biology

**DOI:** 10.1186/gb-2011-12-7-r66

**Published:** 2011-07-25

**Authors:** Gerard Manning, David S Reiner, Tineke Lauwaet, Michael Dacre, Alias Smith, Yufeng Zhai, Staffan Svard, Frances D Gillin

**Affiliations:** 1 Razavi Newman Center for Bioinformatics, The Salk Institute for Biological Studies, 10010 North Torrey Pines Road, La Jolla, CA 92037, USA; 2 Department of Pathology, University of California at San Diego, 214 Dickinson St CTF-C 415, San Diego, CA 92103-8416, USA; 3 Department of Microbiology, Tumor and Cell Biology (MTC), Nobels väg 16, KI Solna Campus, Karolinska Institutet, Box 280, SE-171 77, Stockholm, Sweden; 4 Current address: Proveri Inc., 10835 Road to the Cure, Suite 150, San Diego, CA 92121, USA; 5 Department of Cell and Molecular Biology, Uppsala University, BMC, Box 596, SE-75124, Uppsala, Sweden

## Abstract

**Background:**

The major human intestinal pathogen *Giardia lamblia *is a very early branching eukaryote with a minimal genome of broad evolutionary and biological interest.

**Results:**

To explore early kinase evolution and regulation of *Giardia *biology, we cataloged the kinomes of three sequenced strains. Comparison with published kinomes and those of the excavates *Trichomonas vaginalis *and *Leishmania major *shows that *Giardia*'s 80 core kinases constitute the smallest known core kinome of any eukaryote that can be grown in pure culture, reflecting both its early origin and secondary gene loss. Kinase losses in DNA repair, mitochondrial function, transcription, splicing, and stress response reflect this reduced genome, while the presence of other kinases helps define the kinome of the last common eukaryotic ancestor. Immunofluorescence analysis shows abundant phospho-staining in trophozoites, with phosphotyrosine abundant in the nuclei and phosphothreonine and phosphoserine in distinct cytoskeletal organelles. The Nek kinase family has been massively expanded, accounting for 198 of the 278 protein kinases in *Giardia*. Most Neks are catalytically inactive, have very divergent sequences and undergo extensive duplication and loss between strains. Many Neks are highly induced during development. We localized four catalytically active Neks to distinct parts of the cytoskeleton and one inactive Nek to the cytoplasm.

**Conclusions:**

The reduced kinome of *Giardia *sheds new light on early kinase evolution, and its highly divergent sequences add to the definition of individual kinase families as well as offering specific drug targets. *Giardia*'s massive Nek expansion may reflect its distinctive lifestyle, biphasic life cycle and complex cytoskeleton.

## Background

Protein kinases modulate most cellular pathways, particularly in the co-ordination of complex cellular processes and in response to environmental signals. About 2% of genes in most eukaryotes encode kinases, and these kinases phosphorylate over 30% of the proteome [1]. Kinases regulate the activity, localization and turnover of their substrates. Most kinases have dozens of substrates, and operate in complex, multi-kinase cascades. Hence, organisms with reduced kinomes can provide simple model systems to dissect kinase signaling.

**Figure 1:**
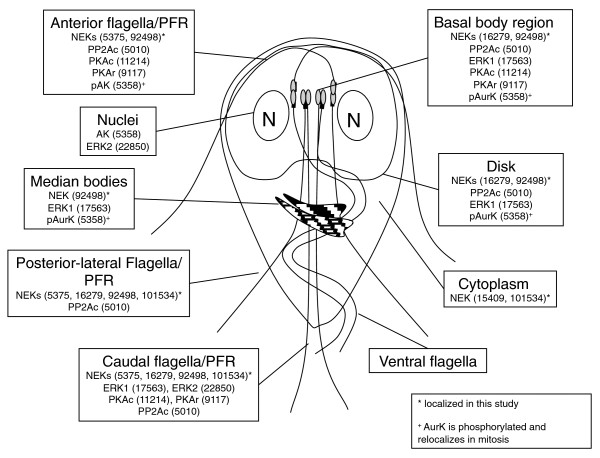
**Cartoon of an interphase *Giardia *trophozoite showing kinases that have been immunolocalized to date**. The localizations of previously described kinases, PP2A and the Nek kinases reported in this study are shown. In most cases, the kinases localize to the intracellular flagella-associated paraflagellar dense rods (PFRs), rather than to the axonemes. (Modified from [64].)

The unicellular human gut parasite *Giardia lamblia *cycles between a dormant cyst stage and a virulent trophozoite, both of which are adapted to survival in different inhospitable environments [2]. The life cycle starts with the ingestion of the cyst by a vertebrate host. Exposure to gastric acid during passage through the host stomach triggers excystation and the parasite emerges in the small intestine after stimulation by intestinal factors [3,4]. The excyzoite [5] quickly divides into two equivalent binucleate trophozoites that attach to and colonize the small intestine. Trophozoites carried downstream by the flow of intestinal fluid differentiate into dormant quadrinucleate cysts. Cysts are passed in the feces, and can survive for months in cold water until they are ingested by a new host. Trophozoites are half-pear shaped and are characterized by four pairs of flagella, a ventral attachment disk and a median body (Figure 1). Each pair of flagella has a distinct beating pattern and likely has dedicated functions in swimming and attachment [6,7].

The recent genome sequencing of strains from three assemblages (broadly equivalent to subspecies) of *Giardia lamblia *(syn. *intestinalis*) [8-10] revealed a compact genome of approximately 6,500 ORFs that is highly divergent in sequence from other eukaryotes. Many conserved pathways have substantially fewer components than in similarly sized genomes [8]. Its minimal genome and the ability to culture and induce its complex life and cell cycle *in vitro *make *Giardia *an appealing model for studying the signaling underlying entry into and emergence from dormancy in a pathogen.

Few kinases and phosphorylation patterns have been studied in *Giardia *(Table 1) [11,12]. Functional studies [13-16] suggest that regulation of protein phosphorylation by kinases and phosphatases plays a central role in modulating the dramatic remodeling of the parasite's morphology as it cycles between the dormant infectious cyst and the motile, virulent trophozoite (Table 1). Many of the known signaling proteins localize to cytoskeletal structures unique to *Giardia*, which may confer functional specificity (Figure 1).

**Table 1:** *Giardia *protein and lipid kinases and protein phosphatases published to date

Kinase	ORF ID	Localization (immunofluorescence, tag or specific antibody)	Protein expression (immunoblot)	Reported function	Reference
Aurora kinase (AurK)	5358	Interphase: nuclei. Mitosis: activated by phosphorylation. pAurK: centrosomes, spindle, anterior PFR, median body, parent attachment disk	Constant in encystation	Mitosis, cell cycle (inhibitors)	[52]
PKAc	11214	Basal bodies, anterior, caudal PFR. Encystation: basal bodies only	Constant in encystation	Encystation, excystation (inhibitors)	[13,14]
PKAr	9117	Basal bodies, anterior, caudal PFR. Encystation: greatly decreased	Strongly decreased in encystation	Decreases activity of PKAc	[14]
Akt (PKB)	11364				[47]
ERK1	17563	Median body, outer edge of attachment disk	Gradually reduced during encystation	Reduced activity in encystation	[16]
ERK2	22850	Nuclei, caudal flagella. Encystation: cytoplasmic, punctate	Not greatly changed in encystation	Reduced activity in encystation	[16]
PI3K1	14855			Growth (inhibitors)	[48,49]
PI3K2	17406			Growth (inhibitors)	[48,49]
PI4K	16558			Growth (inhibitors)	[48]
PKA	86444	[Reported as a PKCβ]			[24]
TOR	35180				[48,50]
Protein phosphatase					
PP2Ac	5010	Basal bodies, anterior, caudal, posterior-lateral PFR. Encystation: localization to anterior PFR lost, cyst wall	Highest in cysts, stage I excystation	Encystation, excystation (inhibitor, antisense)	[15]

PFR, paraflagellar rod. See Additional file 4 for definitions.

Protein kinases are well-studied in other organisms, control most aspects of cellular functions, and are proven therapeutic targets. Hence, analysis of the *Giardia *kinome may give valuable insight into this parasite's biology and the evolution of signaling.

## Results and discussion

We cataloged the *Giardia *kinome using hidden Markov model (HMM) profiles and Blast searches of genomic and EST sequences from three sequenced strains: two established human pathogens, WB (assemblage A) [8] and GS (assemblage B) [9], that appear to span the divergence of isolates infectious to humans, and a recently isolated porcine strain, P15 (assemblage E) [10]. Despite their shared genus name, these genomes are quite divergent, with an average of 90% protein sequence identity between WB and P15, and approximately 79% between these two strains and GS [10].

We found 278 protein kinases in the WB strain (Table 2; Additional file 1), 272 in GS, and 286 in P15, using release 2.3 of the *Giardia *genomes [17]. These include 46 new gene predictions and 86 sequences not previously annotated as kinases. We also extend 30 fragmentary gene predictions from WB to longer pseudogene sequences. Remarkably, over 70% of the kinome belongs to a huge expansion of one family, the Nek kinases. Since these have so many unusual characteristics, we will refer to the 80 non-Nek kinases as the core kinome and consider the Nek expansion separately.

**Table 2:** Summary of *Giardia *kinome classification

Group	Family	Subfamily	Count	ORF ID	Notes
**Primordial kinases in *Giardia *strain WB (core kinome plus Nek1)**					
AGC	Akt		1	11364	Metabolic rate control
AGC	NDR	NDR-unclassified	2	8587, novel	Mitotic exit, morphology, centrosomes
AGC	PDK1		1	113522	Lipid signaling, AGC master kinase
AGC	PKA		2	11214, 86444	cAMP responsive kinase
AGC	PTF	FPK	1	221692	Potential flippase kinase
CAMK	CAMK1		1	11178	Calcium-dependent signaling
CAMK	CAMKL	AMPK	3	14364, 16034, 17566	Energy metabolism
CK1	CK1	CK1-D	1	7537	Absent from ciliates and plants
CMGC	CDK	CDC2	3	15397, 8037, 9422	Master kinase of cell cycle
CMGC	CDK	CDK5	1	16802	Non-cell cycle CDK
CMGC	CDKL		1	96616	Functions unknown
CMGC	CK2		1	27520	Diverse functions, hundreds of substrates
CMGC	CLK		1	92741	Splicing and other functions
CMGC	DYRK	DYRK1	1	101850	Not in ciliates, *Trichomonas*, or moss
CMGC	DYRK	DYRK2	3	137695, 17417, 17558	Varied functions
CMGC	GSK		2	17625, 9116	Glycogen synthase kinase 3. Diverse functions
CMGC	MAPK	ERK1	1	17563	Canonical MAPK pathway
CMGC	MAPK	ERK7	1	22850	Variant MAPK gene
CMGC	RCK	MAK	2	14172, 6700	Meiosis, flagella
CMGC	RCK	MOK	1	14004	Flagellar regulation
CMGC	SRPK		1	17335	Splicing
Other	Aur		1	5358	Mitotic kinase
Other	Bud32		1	16796	Telomere associated (KEOPS complex)
Other	CAMKK		1	96363	CAMK kinase
Other	CDC7		1	112076	Cell cycle
Other	IKS		1	137730	Not in ciliates or moss
Other	NAK	NAK-unclassified	2	12223, 2583	Varied functions
Other	NEK	NEK1	1	137719	Flagellar and centrosomal functions. Only Nek with clear non-excavate orthologs
Other	PEK	GCN2	1	12089	Response to amino acid starvation
Other	PLK	PLK1	1	104150	Mitotic kinase. Lost in plants
Other	SCY1		1	8805	Cryptic functions
Other	TTK		1	4405	Not in ciliates or moss
Other	ULK	Fused	1	17368	Varied functions
Other	ULK	ULK	1	103838	Autophagy
Other	Uni1		1	16436	Uncharacterized. Lost in plants, fungi, animals
Other	VPS15		1	113456	Vesicular transport, autophagy
Other	WEE	WEE-unclassified	1	115572	Key cell cycle kinase
Other	WNK		1	90343	Osmotic balance
PKL	PIK	FRAP	1	35180	Metabolic rate control (mTOR/TOR)
PKL	PIK	PIK-unclassified	1	16805	Weakly similar to ATR, but may be a lipid kinase
PKL	RIO	RIO1	1	17449	Ribosome biogenesis
PKL	RIO	RIO2	1	5811	Ribosome biogenesis
STE	STE11	CDC15	2	16834, 6199	Functions in mitotic exit; lost in plants and holozoans
STE	STE11	STE11-unclassified	1	1656	MAP kinase kinase kinase
STE	STE20	FRAY	1	10609	Not in ciliates, usually co-occurs with Wnk
STE	STE20	MST	1	15514	NDR kinase
STE	STE20	PAKA	1	2796	Transduces membrane signaling from small GTPases
STE	STE20	YSK	1	14436	Universal STE20 kinase
STE	STE7	MEK1	1	22165	MAP kinase kinase
					
***Giardia*-specific classes and unique kinases**					
Other	Nek	Nek-GL1	11	Table S1^a^	
Other	Nek	Nek-GL2	3	Table S1^a^	
Other	Nek	Nek-GL3	4	Table S1^a^	
Other	Nek	Nek-GL4	32	Table S1^a^	
Other	Nek	Nek-Unclassified	147	Table S1^a^	
CMGC	CMGC-GL1		2	17139, 21116	Divergent pair of CMGC-like kinases
Other	Other-GL1		3	17392, 17378, 6624	Trio of kinases with no specific homologs
Other	Other-unique		8	Table S1^a^	Kinases with no specific homologs
CMGC	CDK	CDK-unclassified	3	11290, 4191, 14578	Divergent cyclin-dependent kinase
CAMK	CAMK-unique		1	13852	Divergent CAMK group member
CAMK	CAMKL	CAMKL-unclassified	2	14661, 9487	Divergent CAMKL family member
					
**Non-protein kinases from PKL**					
PKL	CAK	ChoK	1	4596	Choline and aminoglycoside kinase
PKL	CAK	FruK	1	2969	Fructosamine kinase
PKL	PIK	PI3K	2	14855, 17406	Phosphatidyl inositol 3' kinase
PKL	PIK	PI4K	1	16558	Phosphatidyl inositol 4' kinase
					
**Basal kinases found in *Trichomonas*, but not *Giardia***					
AGC	MAST	MAST			Microtubule-associated serine kinases. Lost in fungi
Atypical	TAF1				Basal transcriptional machinery, TFIID subunit
CAMK	CDPK				Calcium-dependent protein kinase. Lost from unikonts
CK1	TTBK				Tau tubulin kinase. Found in unikonts, some chromalveolates, and excavates
CMGC	CDK	CDK7			Transcription initiation and DNA repair: subunit of TFIIH
CMGC	CDK	CDK12 (CRK7)			Phosphorylates CTD of RNA polymerase II
CMGC	DYRK	YAK			Lost in metazoans. Possible function in splicing
Other	TLK				DNA break repair. Lost in fungi, *Dictyostelium*
PKL	PIK	ATM			DNA break repair
PKL	PIK	ATR			DNA break repair
CMGC	CDK	CDK20 (CCRK)			Cilium-associated, CDK-activating kinase. Found in unikonts, algae, and *Trichomonas*
TKL					Diverse group related to tyrosine kinases
					
**Basal kinases found in *Leishmania *but not *Giardia *or *Trichomonas***					
PKL	ABC1	ABC1-A			Mitochondrial kinase
PKL	ABC1	ABC1-B			Mitochondrial kinase
PKL	ABC1	ABC1-C			Mitochondrial kinase
HisK	PDHK	BCKDK			Mitochondrial kinase
HisK	PDHK	PDHK			Mitochondrial kinase
CMGC	DYRK	DYRKP			Splicing? Also lost in animals, fungi, *Dictyostelium*
PKL	PIK	DNAPK			DNA break repair. Absent from fungi, nematodes, insects, some plants
					
**Basal kinases not found in excavates**					
Other	IRE				Endoplasmic reticulum unfolded protein response
Other	PEK	PEK			Endoplasmic reticulum unfolded protein response. Absent from ciliates
Other	NAK	MPSK			Secretory pathway function. Absent from ciliates
Other	BUB				Mitotic spindle checkpoint. Absent from ciliates
CMGC	CDK	CDK8			Phosphorylates CTD of RNA polymerase II
CMGC	CDK	CDK11			Mitotic spindle function? Absent from fungi
CMGC	DYRK	PRP4			Splicing. Lost in fungi
PKL	PIK	SMG1			Nonsense-mediated decay of spliced transcripts. Absent from ciliates
HisK	HisK				Histidine kinases. Absent from metazoans
PKL	Alpha	VWL			Functions unknown. Absent from metazoans
AGC	PKG				cGMP-activated protein kinase. Absent from fungi, *Dictyostelium*
PKL	ABC1	ABC1-D			Mitochondrial kinase. Absent from ciliates
Atypical	G11				Function unknown. Absent from ciliates
Other	PLK	SAK			Mitotic kinase. Absent from plants
Other	Haspin				Functions in mitosis. Absent from ciliates
AGC	RSK				Ribosomal S6 kinase. Excavates lack conserved substrates sites in tail of ribosomal protein S6
CAMK	CAMKL	MARK			Microtubule affinity-regulating kinase. Absent from plants
					
**Other kinases shared between excavates and one other ancient group**					
CAMK	CAMKL	LKB			Activator of other CAMKL kinases. Found in excavates and unikonts, lost in *Giardia *and *L. major*
CAMK	CAMKL	CIPK	1	16235	Found in plants and excavates. CBL-interacting protein kinases
CK1	CK1	CK1y			Found in plants and *Trichomonas*

^a^See Additional file 1 for details. BCKDK, branched chain ketoacid dehydrogenase kinase; mTOR, mammalian target of rapamycin; PDHK, pyruvate dehydrogenase kinase; RSK, ribosomal S6 kinase; TLK, Tousled-like kinase; TOR, target of rapamycin. See Additional file 4 for definitions of other proteins.

### The core kinome

The core kinome of 80 kinases is completely conserved between the three genomes. Sixty-one core kinases can be classified into 49 distinct classes (families or subfamilies) that are conserved in many other eukaryotes [18-23]; the remaining 19 include 5 in two small *Giardia*-specific families, and 14 with no close homologs (Table 2; Additional file 1). *Giardia *sequences are typically the most divergent of any within their families: comparison of a set of nine universally conserved kinase domain orthologs from human to various deep-branching lineages showed an average sequence identity of only 40% for *Giardia*, compared with 46% for the related excavate *Trichomonas vaginalis*, and 46 to 50% for other deep-branching lineages (ciliates, plants, fungi) (Additional file 2). This indicates that *Giardia *sequences are remarkably divergent, even for an early-branching lineage, and provides a useful resource to study the limits of how sequences can vary while still retaining their family-specific functions. Thus, *Giardia *encodes the smallest and most sequence-divergent of studied eukaryotic kinomes, other than those of parasites that have not been cultured axenically. No core kinome class has more than three members in *Giardia*, suggesting a lack of recent duplication and expansion into specialized functions.

Two previously predicted kinases could not be found: a protein kinase C (PKC) was inferred earlier by reactivity to antibodies against mammalian PKCs and by PKC-selective inhibitors [24], but no clear PKC homolog is seen in the genome sequence. Similarly, although an insulin-like growth factor receptor (IGFR) kinase was inferred by antibody binding and association with phosphotyrosine [25], we could not find an IGFR in the genomes of *Giardia *or any other protist.

### Evolutionary origin and functional repertoire of the *Giardia *kinome

**Figure 2:**
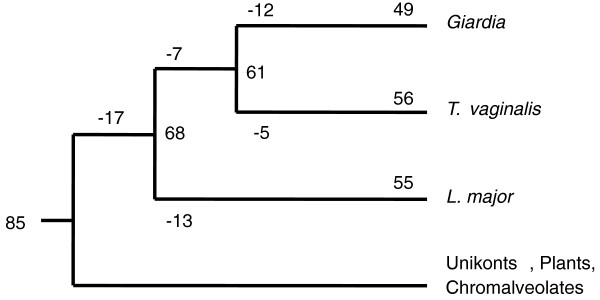
**Loss of kinases in the lineage leading to *Giardia***. Sixty-seven kinase classes are shared between one of the three excavates *Giardia*, *Trichomonas vaginalis *and *Leishmania major *and at least two other major clades (unikonts, plants or chromalveolates). An additional 17 kinases are missing from all three excavates but found in at least two of the outgroups and may be excavate losses (giving a primordial kinome of 84 kinase classes) or later eukaryotic inventions if excavates were indeed the earliest-diverging lineage. Kinase classes are listed in Table 2.

To probe the origin of the *Giardia *kinome, we annotated the kinomes of two other excavates, *Trichomonas vaginalis *[26] and *Leishmania major *[27] (Additional file 3). The excavates are one of about six anciently diverged 'supergroups' of eukaryotes, whose relationship to each other is uncertain [28]. Excavates include free-living, symbiotic, and parasitic protists, many flagellated and often with reduced mitochondria. Comparison of the three excavate kinomes predicts a rich kinome of 68 distinct kinases in their common ancestor, with substantial losses of core kinases in extant species, possibly due to their reduced parasitic lifestyles [29] (Figure 2, Table 2). These losses provide a valuable model to explore the effect of gene deletion on pathway evolution and organismal biology. All three excavates lack 17 kinase classes found in at least two other major eukaryotic groups (unikonts, plants, chromalveolates), suggesting a very early divergence of the excavates [30] and/or even more losses across the entire clade. This suggests that the common ancestor of extant eukaryotes had 85 different kinase classes (or 68 if excavates are the earliest-diverging clade), substantially more than previous estimates [19,20], and attesting to the many diverse conserved roles of kinases. Several noteworthy themes emerge from these losses (Table 2; see below).

### Distinctive patterns of kinase losses in the *Giardia *lineage

Five of the seven ancient kinases lost from *Giardia *and *T. vaginalis*, but found in *L. major*, are mitochondrial kinases (ABC1-A, -B, -C, PDHK, BCKDK), consistent with the degeneration of the mitochondrion to a mitosome or hydrogenosome in these largely anaerobic species [31]. A separate degeneration occurred in some amoebozoa, and accordingly, these kinases are also secondarily lost from *Entamoeba histolytica *(GM, unpublished). The other two are likely involved in DNA repair and splicing (see below). The 17 kinases found in other early branching lineages but absent from excavates include IRE1 and PEK, which mediate endoplasmic reticulum stress responses, supporting the observed lack of a physiological unfolded protein response in *Giardia *[32] (see Additional file 4 for definitions of kinase classes discussed in the text). *Giardia *has unusual dual mitotic spindles [33], and all three excavates also lack the spindle-associated kinases BUB and cyclin-dependent kinase (CDK)11. They all also lack the mitosis-associated kinases SAK and Haspin, and their lack of a ribosomal S6 kinase (RSK) correlates with the lack of a regulatory substrate serine in the tail of ribosomal protein S6 in all excavates. Genes lost only from *Giardia *include three encoding DNA repair kinases (ATR, ATM, TLK) and two RNA polymerase kinases (CDK7, CDK12). Despite having an elaborate microtubule cytoskeleton, *Giardia *has lost the microtubule-associated kinases MAST and TTBK (Tau tubulin kinase), while microtubule affinity-regulating kinase (MARK) is missing from all excavates. Splicing and RNA-linked kinases DYRKP, YAK, PRP4, and SMG1, and basal transcription factor kinases TAF1 and CDK8 are also lost in different patterns within the excavates, suggesting gradual divergence or reduction in the regulation of these processes.

### Losses of DNA repair kinases may explain sensitivity to radiation and chemical DNA damage

The PIKKs (phosphatidyl inositol 3' kinase-related kinases) ATM, ATR, and DNAPK are involved in recognition and repair of DNA breaks [34]. Deletions of these in several organisms lead to increased radiation and mutagen sensitivity. *Giardia *is the only eukaryote known to lack all three, though it has one gene (GK009) with very weak similarity to the ATR and ATM kinase domains, yet lacks their conserved accessory domains. *Giardia *also lacks the Chk1 and Chk2 checkpoint kinases that are activated by ATM and ATR, and the downstream TLK kinases [35]. ATM, ATR, and TLK are all found in *T. vaginalis*. *Giardia *does have homologs of other DNA break repair proteins, including MRE11 and RAD50 of the MRN complex, suggesting that aspects of DNA break repair may be functional, but perhaps recognized by a divergent mechanism. *Giardia *has a single histone H2A with a H2Ax-like ATM/ATR substrate site. Induction of double-stranded DNA breaks in trophozoites results in anti-phospho-H2A antibody staining [36]. This suggests that some ATM/ATR-like kinase activity may be present, possibly acting through GK009. *Giardia *also lacks both DNAPK and its binding partners, Ku70 and Ku80, indicating that DNA break repair may be severely diminished or divergent in *Giardia*. This lack of DNA repair kinases correlates with the reported sensitivity of *Giardia *cysts to low doses of UV light and inability to repair DNA breaks [37].

### Transcription and splicing kinases

Several CDK family members control RNA polymerase II by phosphorylation of a heptad repeat region in its carboxy-terminal domain (CTD) in plants and animals. These include CDK7, CDK8, CDK9 [38] and CDK12 (CRK7) [39]. Some protists, including ciliates and trypanosomes, lack both the heptad repeat of RNA polymerase II and CDK7/8/9, but retain CDK12, and several have many Ser-Pro (SP) motifs in the CTD, suggesting that CDK12 may phosphorylate this tail. *T. vaginalis *retains CDK7 and CDK12 and has 19 SP sites in the CTD, while *Giardia *has only two SP sites and has lost both kinases. CDK12 has also been associated with splicing, which is common in ciliates and trypanosomes, but very rare in *Giardia*. PRP4 is another splicing-associated kinase lost from *Giardia*, but other splicing kinases (SRPK, DYRK1, DYRK2) are retained, suggesting that these may have different functions, or be retained for use in the rare cases of *Giardia *splicing [8].

*Giardia *also lacks TAF1, an atypical kinase constituent of the general transcription factor TFIID that is known to phosphorylate Ser33 of histone H2B. *Giardia *H2B lacks this serine, and none of the other 13 subunits of TFIID have been identified [40]. TAF1 and several other TFIID complex members are found in *T. vaginalis*, suggesting loss of this complex from *Giardia*.

### Histidine and tyrosine phosphorylation

**Figure 3:**
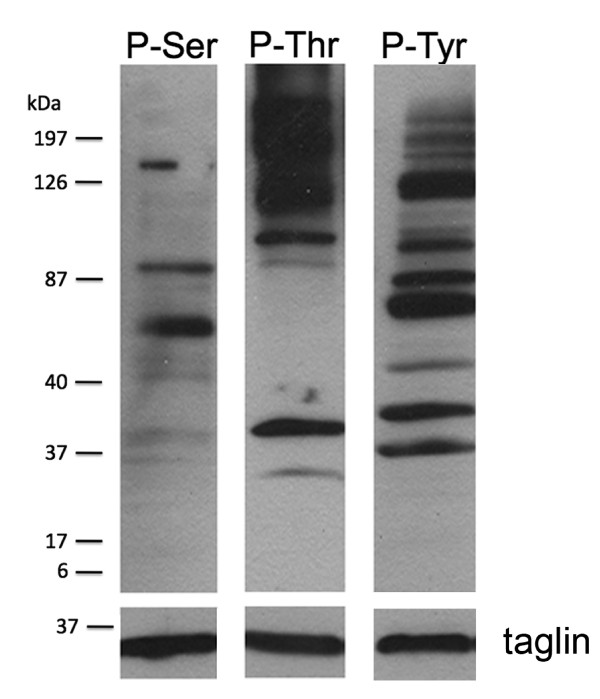
**Distribution of serine, threonine and tyrosine phosphorylated proteins**. Western blot of total *Giardia *trophozoite lysates individually labeled with antibodies recognizing phosphoserine (P-Ser), phosphothreonine (P-Thr), or phosphotyrosine (P-Tyr). The taglin loading control is shown at the bottom of the figure.

**Figure 4:**
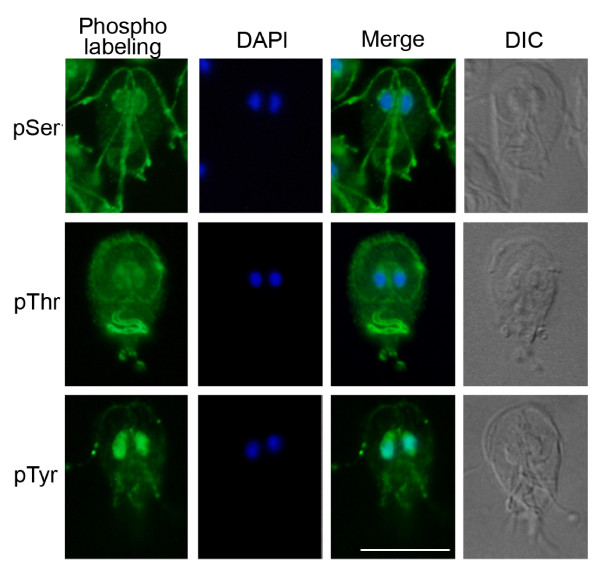
**Immunolocalization of serine, threonine and tyrosine phosphorylated proteins in *Giardia *trophozoites**. Interphase trophozoites were labeled with antibodies against phosphoserine (pSer), phosphothreonine (pThr), or phosphotyrosine (pTyr). Phospholabeling is shown in green, nuclei are labeled with DAPI and a merge image shows overlay between the two stains. Morphology is shown in a differential interference contrast (DIC) image of each trophozoite. Scale bar = 10 μm.

Unlike plants and most protists, *Giardia *lacks classical histidine kinases. Tyrosine phosphorylation in *Giardia *trophozoites can be seen by western blot (Figure 3), [11], proteomics (TL, FG, unpublished), and immunofluorescence (Figure 4). However, we found no classical tyrosine kinases (TK group) or members of the related tyrosine kinase-like (TKL) group. A number of other serine-threonine-like kinases have been reported to phosphorylate tyrosine, including Wee1 (cell cycle), MAP2K (though only acting on the MAPK activation loop), and TLK, while DYRK and glycogen synthase kinase (GSK) family kinases can autophosphorylate on tyrosine [41]. Phosphoproteomic profiling of the excavate *Trypanosoma brucei *shows that more than half of the recorded phosphotyrosine (pTyr) phosphorylation events were found on these kinases [42]. *Giardia *has one Wee, one MAP2K, one GSK, and four DYRK family kinases. *Giardia *has no SH2 or PTB phosphotyrosine-binding domains, supporting the lack of a phosphotyrosine signaling system as has been inferred in animals, plants, and *Dictyostelium *[20,43]. By contrast, several proteins with putative phosphoserine or phosphothreonine binding domains are present: two clear forkhead-associated (FHA) domains, one 14-3-3, one WW and over 250 WD40 domains. Of these, only the 14-3-3 protein has been characterized and shown to bind phosphopeptides [44]. *Saccharomyces cerevisiae *also lacks TK and TKL group kinases, but shows substantial tyrosine phosphorylation by phosphoproteomics [1]. These data from both *Saccharomyces *and *Giardia *suggest that dual-specificity or undetected tyrosine kinases may be more important than previously thought.

### Accessory domains are reduced or divergent

Most kinases from other genomes have additional domains that help in regulation, localization, or scaffolding. Many core *Giardia *kinases lack detectable accessory domains. However, the domains that are present correlate well with conserved family-characteristic domains [18]: polo boxes in PLK family kinases; PBD/CRIB domains in PakA; HEAT, FAT and FATC domains in TOR; and pkinase_C in one PKA and one NDR kinase (Additional file 1; see Additional file 4 for definitions of domains). Cryptic PH domains are seen in Akt and PDK1, and the characteristic pkinase_C domain is absent from other AGC kinases, although this can be difficult to detect on such remote sequences. Several other kinases have regions of novel sequence outside of the kinase domain that may be orthologous domains too divergent in sequence to be detectable. No kinase has a clear signal peptide, and only four are predicted to have transmembrane domains. This is consistent with the observed false positive rate for predicting these regions, suggesting that *Giardia *has no receptor kinases. Other unrelated parasitic protists, including *Entamoeba histolytica*, have a rich complement of receptor kinases [45]. The Nek kinases are highly enriched for ankyrin repeats and coiled-coil regions (see below).

### Catalytically dead kinases

In most kinomes, about 10% of kinases lack critical catalytic residues (K72, D166, D184) and are likely to be catalytically inactive, yet may retain signaling functions as scaffolds or kinase substrates [46]. In the WB strain, 10% (8 of 80) of the core kinome and 71% (139 of 195) of Neks lack one or more of these three key residues and are likely to be inactive (Additional file 1). The eight inactive core kinases include Scyl, whose orthologs are all inactive, and Ulk, which has some inactive homologs in other species. The functions of both families in any organism remain obscure. Four pseudokinases are highly divergent proteins specific to *Giardia*; some might have cryptic active sites that could not be found by alignment to other kinases.

### AGC signaling

The AGC kinase group (PKA/PKG/PKC kinases) mediates a wide variety of intracellular signals, including nutrient, phospholipid and extracellular signal responses. *Giardia *has seven AGC kinases, including a very divergent PDK1, Akt (GiPKB) [47], two PKAs (cyclic AMP-regulated kinases) [13,14], a lipid flippase kinase (FPK) and two NDR kinases. The *Akt *and *PDK1 *genes are particularly divergent, but are partially validated by the presence of weakly predicted phospholipid-binding PH domains, and a likely PDK1 phosphorylation site that is seen in the activation loop of all *Giardia *AGC kinases. A possible PDK1-binding 'hydrophobic motif' is found in Akt (FKDF) and in one NDR kinase (YTYRA), but not in other AGC kinases, and no neighboring phosphorylation site is seen.

Cyclic AMP-dependent signaling is confirmed by the presence of two PKA catalytic subunits (Additional file 1), one regulatory subunit (Orf_9117 in GiardiaDB) [14], and one homolog (Orf_14367) of adenylate and guanylate cyclases. No clear AKAP (A kinase anchoring protein) was found. In many organisms, including *Giardia*, PKA localizes to the basal bodies/centrosomes [13]. In addition, both the catalytic (PKAc) and regulatory (PKAr) subunits localize to the paraflagellar rods rather than the flagellar axonemes [13,14] (Table 1, Figure 1). PKAc and PKAr localization to the basal bodies is constitutive, while their distribution to the paraflagellar rods is influenced by external stimuli, such as growth factors, encystation stimuli and cAMP levels [13]. Inhibitor studies indicate that PKAc activity is also required for the cellular awakening of excystation [13].

### Phospholipid signaling

The two *Giardia *phosphatidyl inositol kinases PI3K and one PI4K have been cloned and are expressed in trophozoites and encysting cells [48-50]. As in other species, PI3K likely relays signals from transmembrane receptors by activation of the protein kinase PDK1 to phosphorylate the survival kinase Akt and several other AGC group kinases, as well as the PI3K-like protein kinase TOR, which modulates energy level responses. This suggests that *Giardia *has intact phospholipid signaling pathways coupled to non-kinase receptors.

### MAPK cascade

The MAPK cascade consists of a relay of up to four kinases that phosphorylate and activate each other, usually to transmit signals from the cell surface to the nucleus. The prototypical MAPK cascade involves the Erk MAPK, which is phosphorylated on both serine and tyrosine by a MAP2K (MEK, MKK, Ste7), which in turn is serine phosphorylated by a MAP3K (MEKK, Ste11), and that by a MAP4K. MAP2K, some MAP3Ks, and MAP4K make up the three families of the STE group of kinases, while Raf and MLK MAP3Ks are from the TKL group. All four kinase classes are found in all analyzed eukaryotic kinomes, apart from *Plasmodium *[51]. *Giardia *has one canonical Erk (Erk1), and a member of the distinct Erk7 MAPK subfamily, called Erk2 [16]. Both genes have the MAP2K dual phosphorylation motif (T[DE]Y sequence). We found a single MAP2K, along with three MAP3K and four MAP4K genes, one each from the primordial FRAY, MST, PAKA and YSK subfamilies. The single MAP2K indicates either that all the upstream kinases funnel though this single gene, or that there are alternative pathways that bypass MAP2K, for which *Giardia *may be a tractable model. Two of the three MAP3Ks are homologs of *S. cerevisiae *Cdc15, involved in the mitotic exit network and cytokinesis. These have orthologs in plants and other basal eukaryotes, but not in animals. The distinct functions of Erk1 and Erk2 are highlighted by their localization: in vegetative trophozoites, Erk1 was found in the disk and median body while Erk2 was in the nuclei and caudal flagella [16] (Figure 1). During encystation, their expression levels remained the same, but their phosphorylation and kinase activity were reduced and Erk2 became more cytoplasmic (Table 1).

### Cell cycle

*Giardia *has a full complement of basic cell cycle kinases (Table 2). These include three CDK1/CDC2 kinases, along with three mitotic (A/B) cyclins, a putative CDK5, three unclassifiable CDKs and two unclassifiable cyclin-like genes, as well as a Wee1 homolog. We also found single copies of the Aurora (AurK) and Polo (PLK) mitotic kinases, which are activated in M phase and involved in centrosome or kinetochore function, spindle assembly and cytokinesis. *Giardia *AurK is exclusively nuclear during interphase. During mitotic prophase, it is activated by phosphorylation and migrates to the mitotic spindle poles, as well as to the median bodies and anterior paraflagellar rods (PFRs; Figure 1) [52]. Beginning in metaphase, pAurK localizes to the parental attachment disk, which persists until the daughter disks are developed. AurK inhibitors decreased growth and led to abnormal cytokinesis. Thus, AurK has a *Giardia*-specific localization and likely function in addition to its universal function and location in the mitotic spindle. In mammalian cells, Aur kinase is centrosomal, but interestingly, in *Chlamydomonas *gametes, it is localized to the flagellar tips or adhesion sites [53].

### Expansion and divergence of the *Giardia *Nek kinase family

**Figure 5:**
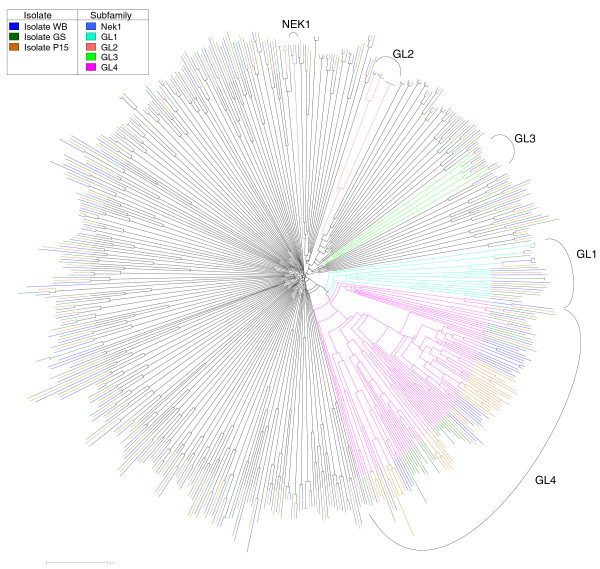
**Phylogenetic tree of Nek kinase domain sequences, from alignment S1**. Most kinases have close orthologs between the strains (WB is shown in dark blue, GS in green, and P15 in orange), but have very little similarity to orthologs. Deeper branches of defined subfamilies are also labeled by arcs and colored: Nek1 (light blue), GL1 (cyan), GL2 (light red), GL3 (bright green), and GL4 (purple). See Additional file 5 for an expanded and labeled version of this tree.

The Nek kinase family is universal in eukaryotes, and its members regulate entry to mitosis [54] and flagellum length [55,56]. The Nek family is expanded in both ciliates and excavates, with 40 genes in *Tetrahymena *and 11 to 25 in trypanosomes [27,57], compared with only 11 in humans and one in yeast. *Giardia *strain WB has a massive 198 Neks, making up 71% of its kinome and about 3.7% of the entire proteome. These have remarkably divergent sequences (Figure 5; Additional file 5); all but 56 have lost critical catalytic residues and are likely pseudokinases, and many show detectable sequence similarity only to other Neks but not to standard kinase domain models. Most retain a number of structural motifs (Additional files 6 and 7), but are so divergent in overall sequence that our count may not be precise.

The Neks are evolutionarily dynamic, accounting for all of the kinase gain and loss between *Giardia *strains. While 99.7% of all 4,570 'core' WB genes are found in strains GS and P15 [10], the Neks are one of four families (along with Protein 21.1, HCMP (high cysteine membrane protein) and VSP (variable/variant surface protein) genes) that are both highly expanded and polymorphic between strains, and may be responsible for strain-specific characteristics. Seventy-nine Neks (30%) are found in only one strain and a further 31 (12%) are found in two but are absent from the third, due to both gene duplication and loss (Additional file 8). Within the Neks, two patterns emerge: most are highly conserved and slowly evolving between strains, while a subset accounts for most of the gene gains and losses.

Of the Neks, 74% (147 of 198 genes in WB) have no close paralogs (labeled 'Nek-Unclassified'). Their average sequence identity to the next closest Nek is only 34% in the kinase domain, and for the most divergent 10% of Neks, this drops to only 20%. This is less than that of orthologous kinase domains between human and *Giardia *(40%), and even less than that of many kinases from different families, implying rapid diversification in sequence and function. However, they are well conserved between strains; 89% (131) have orthologs in all three strains, and their sequences are only slightly less conserved than those of core kinases (average kinase domain identity of 88% between WB and P15, 78% between WB and GS, compared with 92% and 84% for core kinase domains), indicating that these Neks may be quite ancient, rather than very rapidly evolving.

We classified 51 Neks (26% of Neks in WB) into 5 subfamilies, based on kinase domain sequence similarity: Nek1, which is conserved throughout eukaryotes, and GL1 to GL4, which are *Giardia*-specific. GL1 to GL3 are moderately sized subfamilies with 3 to 11 members each. GL4 is dramatically different. It has 32 members in WB, but only 5 of these genes are single copy in each strain. In total, 87 genes across the three strains are not three-way orthologs; 53 of these are found in 10 strain-specific clusters. The rapid turnover of GL4 Neks is further highlighted by our discovery of an additional 30 kinase pseudogenes in the WB strain (these are not counted in the overall kinome), of which 29 are from GL4. Moreover, five pairs of GL4 Neks are very recent duplicates, with over 98% identity within the pairs. In summary, the *Giardia *Nek expansion includes both highly divergent but evolutionarily stable members, small and largely stable families, and the GL4 family, which is turning over at a remarkable rate.

Of the *Giardia *Neks, 67% (133 of 198) have an amino-terminal kinase domain, followed by a variable array of ankyrin repeats (1 to 26 repeats, median of 8), which are not found in any core kinases. They are also evolutionarily mobile, with related members of most subfamilies having gained or lost these repeats. They are divergent in sequence but form a distinctive subclass, characterized by a four amino acid TALM motif at the start and a conserved E at the end (Additional file 9). Most other *Giardia *TALM-ankyrin (TA) repeats are found in members of the poorly described Protein 21.1 family, which have a similar structure to Neks but lack the amino-terminal kinase domain. Both families also have some members with coiled-coil regions and carboxy-terminal RING domains (Additional file 1, Text S1 in Additional file 2), and both are large and evolutionarily dynamic. Our examination of the amino-terminal regions of 21.1 proteins revealed very divergent kinase domains in 20, and cryptic kinase-like domains may exist in other 21.1 proteins that are beyond our limit of confident detection. The TA repeat is largely specific to *Giardia*: 59% of the 3,355 *Giardia *ankyrin repeats have an exact TALM motif, compared with just 2.6% (54 of 2,028) in human and 0.5% (24 of 4,602) in *T. vaginalis*. Curiously, the only other organism with many TA repeats is the mushroom *Coprinopsis cinerea *[58], which has 73 proteins containing 271 TA repeats, though none of them have kinase domains. Some are chromosomally clustered, but their functions are unknown (GM, unpublished).

### Expression and localization of phosphorylated proteins in *Giardia*

**Figure 6:**
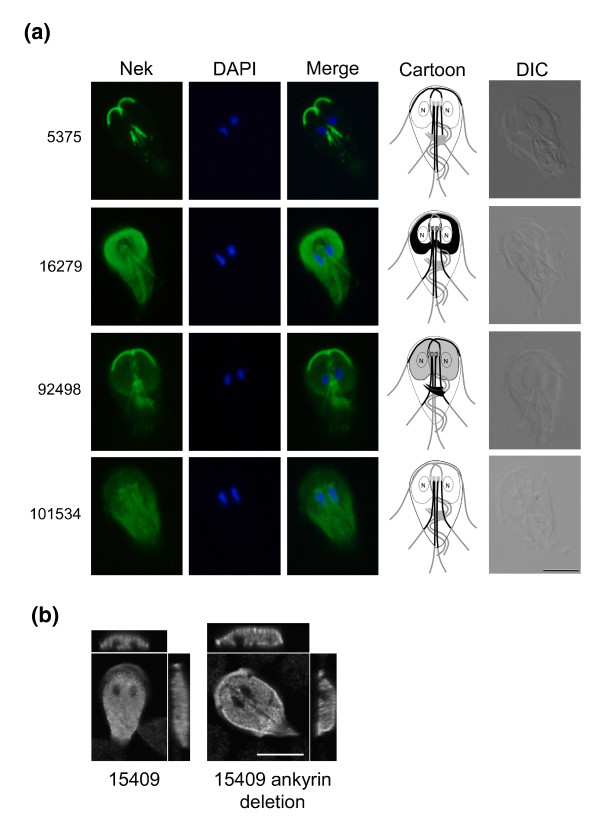
**Immunolocalization of Neks in *Giardia *trophozoites**. **(a) ***Giardia *trophozoites expressing hemagglutinin (HA)-tagged putative active Neks 5375, 16279, 92498, and 101534 were probed with an anti-hemagglutinin-FITC antibody. Each Nek had a distinct cytoskeletal (5375, 16279, 92498, and 101534) or cytoplasmic (101534) localization pattern. In addition to the PFRs, two Neks localized to the ventral attachment disk and the median bodies (16279 and 92498). A trophozoite cartoon further illustrates each specific Nek localization. Nuclei are labeled with DAPI and a differential interference contrast (DIC) image of each trophozoite is shown on the far right. Scale bar = 5 μm. **(b) ***Giardia *trophozoites expressing full-length Nek 15409 and Nek 15409 with the deleted ankyrin repeat were probed with an anti-AU1 antibody and visualized with confocal microscopy. Z-stack images, shown on top and to the right of each image, show that deletion of the ankyrin repeats altered the distribution of 15409 from solely cytoplasmic to a combination of plasma membrane-associated and cytoplasmic. Scale bar = 5 μm.

Signaling proteins often gain specificity by localization close to their targets. This is especially relevant to *Giardia *with its unique cytoskeleton that is remodeled during differentiation. Moreover, the protein kinases characterized to date localize to distinct cytoskeletal structures that are specific to *Giardia *and whose functions remain unclear. We characterized major phosphoproteins by western blot and immunofluorescence, using antibodies against phosphoserine (pSer), phosphothreonine (pThr), and pTyr (Figures 3 and 4). Despite the lack of classical tyrosine kinases in *Giardia*, immunoblots showed strong staining of pTyr, along with pSer and pThr. This corroborates a previous study [11]. Immunofluorescence of *Giardia *trophozoites with the same antibodies revealed distinct patterns for each phospho-amino acid (Figure 4). Consistent with the predicted absence of receptor kinases in *Giardia*, we did not observe staining at the plasma membrane. Strong pSer stain was seen in the intracellular and extracellular portions of three of the four pairs of flagellar axonemes (anterior, posterior-lateral, and caudal; Figure 1) as well as the nuclear envelope, with weaker nuclear and ventral flagellar staining. By contrast, pThr most strongly stained the remaining (ventral) pair of flagella, which beat in a sine wave pattern in both attached and swimming trophozoites [6]. It also stained the rim of the ventral attachment disk and polar regions of the nuclei, possibly the nucleoli [59]. In contrast to the largely cytoskeletal localization of pSer and pThr, pTyr staining was concentrated in the nuclei. It is noteworthy that pSer- and pThr-modified proteins tend to localize to the intracellular and extracellular portions of the flagellar axonemes. In contrast, the Ser/Thr kinases in published studies and two of the four Nek kinases tend to localize to intracellular flagellar-associated structures (Figures 1 and 6; see below). Thus, some of the actual phosphorylation may occur in the basal bodies, and the phosphorylated proteins are then incorporated into the flagella.

### Expression and localization of individual kinases

Gene expression profiling by serial analysis of gene expression (SAGE) [60] confirms expression for 233 kinases, including 156 Neks (Additional file 1). Twenty-seven kinases are categorized as differentially expressed throughout the life cycle, of which 12 kinases, all Neks, were upregulated in trophozoites and encyzoites (encysting cells), and 9 Neks and 4 other kinases were selectively expressed in cysts and excyzoites (excysting cells) (Table 3). Overall, Neks are slightly less likely to be expressed than other genes or kinases, and slightly more likely to be differentially or highly expressed, although the differences are not statistically significant. These data suggest that most Neks are expressed and functional, despite their unusual evolution.

**Table 3:** Differentially expressed kinase transcripts by SAGE

ORF	Group	Family	Subfamily	Orthology	Catalytically active?	SAGE cluster	R	Maximum percentage × 1,000
92498	Other	Nek	Nek-Unclassified	1:1:1	Active	Cysts and excyzoites	9.56	38.5
17625	CMGC	GSK		1:1:1	Active	Cysts and excyzoites	8.30	43.4
8350	Other	Nek	Nek-Unclassified	1:1:1	Active	Cysts and excyzoites	9.10	44
14835	Other	Nek	Nek-Unclassified	1:1:1	Inactive	Cysts and excyzoites	13.68	44
11364	AGC	Akt		1:1:1	Active	Cysts and excyzoites	10.05	63.2
15397	CMGC	CDK	CDC2	1:1:1	Active	Cysts and excyzoites	9.46	71.5
8805	Other	SCY1		1:1:1	Inactive	Cysts and excyzoites	13.93	74.3
17578	Other	Nek	Nek-Unclassified	1:1:1	Inactive	Cysts and excyzoites	13.67	77
91451	Other	Nek	Nek-Unclassified	1:1:1	Active	Cysts and excyzoites	19.69	87.9
95593	Other	Nek	Nek-GL2	1:1:1	Active	Cysts and excyzoites	16.11	92.9
3957	Other	Nek	Nek-Unclassified	1:1:1	Active	Cysts and excyzoites	25.71	109.9
114307	Other	Nek	Nek-GL1	1:1:1	Active	Cysts and excyzoites	24.87	111.5
22451	Other	Nek	Nek-Unclassified	1:1:1	Inactive	Cysts and excyzoites	44.93	255.6
113456	Other	VPS15		1:1:1	Active	Differentiation	9.40	75.2
101307	Other	Nek	Nek-GL1	3:1:1	Active	Trophozoites and encyzoites	10.11	37.8
86934_mod	Other	Nek	Nek-GL1	3:1:1	Active	Trophozoites and encyzoites	10.11	37.8
16943	Other	Nek	Nek-Unclassified	1:1:1	Inactive	Trophozoites and encyzoites	11.98	39.7
3677	Other	Nek	Nek-GL4	1:1:1	Active	Trophozoites and encyzoites	10.31	54
113030	Other	Nek	Nek-GL4	1:1:1	Inactive	Trophozoites and encyzoites	10.14	59.9
5346	Other	Nek	Nek-Unclassified	1:1:1	Active	Trophozoites and encyzoites	10.59	75.6
101534	Other	Nek	Nek-GL4	C	Active	Trophozoites and encyzoites	18.41	75.6
16824	Other	Nek	Nek-Unclassified	1:1:1	Inactive	Trophozoites and encyzoites	8.76	81.5
90343	Other	WNK		1:1:1	Active	Trophozoites and encyzoites	11.42	105.2
114495_mod	Other	Nek	Nek-GL4	C	Pseudogene	Trophozoites and encyzoites	27.04	174.5
24321	Other	Nek	Nek-Unclassified	1:1:1	Inactive	Trophozoites and encyzoites	36.42	213.2
15409	Other	Nek	Nek-Unclassified	1:0:2	Inactive	Trophozoites and encyzoites	35.87	507.4

R is a measure of differential expression, with R = 8 used as a cutoff. Max percentage is the percentage of all SAGE tags belonging to this transcript at its maximum level. ORFs 101307 and 86934_mod are almost identical and share the same SAGE tags. Orthology is the number of orthologous kinases in WB:GS:P15; C means a complex ratio.

To begin to understand the roles of Neks in *Giardia*, we epitope-tagged five Neks under their own promoters. We observed a different localization pattern for each protein (Figure 6). Orf_5375 (Nek-GL2 subfamily) localized prominently to the PFRs of the anterior and posterior-lateral flagella and faintly to the caudal flagella. Orf_16279 (Nek-Unclassified) localized prominently to the outer half of the ventral attachment disk, to the region of the basal bodies and to the caudal and posterior-lateral flagella, but not to the PFRs. Similarly, Orf_92498 (Nek1) localized to the basal bodies/centrosome region in addition to three pairs of PFRs, as well as to the median bodies, disorganized stacks of microtubules unique to *Giardia*, whose functions are unknown [6] (Figure 1). Orf_101534 (Nek-GL4) localized to the posterior-lateral PFR and to the perinuclear regions and cytoplasm. In contrast, Orf_15409 (Nek-Unclassified), which has four ankyrin repeats and is catalytically inactive (Additional file 1), localized diffusely to much of the cytoplasm and to an anterior region that may be plasma membrane associated (Figure 6b). Deletion of the most conserved ankyrin repeat of Orf_15409 (amino acids 351 to 386) resulted in partial relocalization to the plasma membrane (Figure 6b).

The distinct localization of these five Neks likely mirrors their specific functions in the different subcellular compartments. Basal body/centrosomal localization of the conserved Nek1 and the Nek-Unclassified is similar to patterns seen in human (Nek2, Nek6, Nek7, and Nek9), *Chlamydomonas *(Fa2p), *Trypanosoma brucei *(TbNRKC), and *Tetrahymena thermophila *(NRK17p and NRK20p) [55,57,61]. The *Giardia *flagellar basal bodies become spindle poles during mitosis, suggesting that these Neks may be involved in regulating mitotic progression. In other organisms, Neks have also been localized to axonemes. For example, human Nek8 and *Chlamydomonas *Fa2p are found in the proximal region of primary cilia or flagella, respectively, and *Tetrahymena thermophila *NRK1 and NRK30p are located in various types of cilia, with the latter three being involved in regulating flagella/ciliary length [57,62,63]. All four active Neks (Orf_5375, Orf_16279, Orf_92498, and Orf_101534) localize to diverse *Giardia *cytoskeletal structures, and may be involved in regulating flagellar assembly, beat, or cellular attachment [64]. In contrast, the inactive Nek (Orf_15409) is found in the cytoplasm, which may indicate a correlated loss of cytoskeletal association and catalytic activity.

## Conclusions

*Giardia *encodes the simplest known kinome of any eukaryote that can be grown in axenic culture. Some obligate intracellular parasites have even more highly reduced genomes and kinomes (for example, the microsporidian *Encephalitozoon cuniculi *(29 kinases) [65], and *Plasmodium falciparum *(approximately 90) [51]), but are dependent on their hosts for many basic cellular functions, and their lost kinases may be functionally replaced by host kinases.

Protein kinases modulate the vast majority of biological pathways, and this minimal kinome still enables *Giardia *to carry out the broad repertoire of eukaryotic cellular functions needed for its complex life and cell cycles. Our comparison of the *Giardia *kinome to other early branching eukaryotes indicates that the last common ancestor of sequenced eukaryotes had a rich kinome of at least 67 kinase classes, from which *Giardia *has lost at least 18. These include kinases involved in central biological functions, such as DNA repair, transcription, splicing, and mitochondrial metabolism. Exploring how these pathways can function without individual components may help to understand the function of these pathways in more complex organisms.

Other missing kinases, such as those involved in endoplasmic reticulum stress response, are absent from all excavates, and may represent either early losses or reflect that excavates are the earliest branching of eukaryotic lineages. Conversely, *Giardia *retains many ancient kinases (Table 2) whose functions are still largely unexplored, despite their being essential for eukaryotic life.

The *Giardia *kinome is dominated by the expansion of the Nek kinases. The recurrent loss of kinase catalytic function coupled with the conservation of key structural and Nek-specific residues suggest that many Neks maintain a kinase-like fold and serve as scaffolds. The GL4 subfamily is highly dynamic, with most of its members being strain-specific, with loss of catalytic activity even within a single strain, and showing rampant gene duplication and pseudogenization. This high variation rate may underlie important strain differences. However, the rate of pseudogenization also suggests that the rate of duplication of this gene cluster may be enhanced and that at least some copies are under little purifying selection. By contrast, most other Neks are shared between strains and are likely to be anciently diverged, since their paralogs are more remote than orthologs between human and *Giardia*. While a homolog of the universal Nek1 was found, the vast majority of Neks are specific to *Giardia*, and the association with ankyrin repeats is not seen in any other species. The dual mitotic spindles and eight flagella of *Giardia *may explain some of the Nek expansion, but clearly not all of it. Ciliates are also binucleate and have expanded Neks, but no specific orthologs are found between the two clades, apart from Nek1.

We found long runs of a specific class of ankyrin repeat (TALM-Ankyrin: TA) in most Neks. These are likely important for their subcellular localization or protein interactions. While the four active Neks examined had very specific localization and did not contain ankyrin repeats, the deletion within the ankyrin region in Orf_15409 did alter its localization. Several genes annotated as Protein 21.1 are now found to be Neks, and the overall sequence and domain composition suggests that the Neks and Protein 21.1 genes may form a single family with related functions. The other two large, dynamic *Giardia *families (VSP and HCMP) are also related to each other and VSPs undergo antigenic variation [66]. However, the roles and reasons for the expansion and variability of HCMP, 21.1 and Nek remain obscure. The arrays of divergent TA repeats and our results with non-ankyrin-containing Neks indicate that specific subcellular targeting is important for their function, and may allow *Giardia *to regulate complex processes within its single cell by targeting proteins to specific organelles. The Neks constitute a major target for exploration of *Giardia*-specific and strain-specific biology, and their extreme sequence divergence will be useful to explore the sequence limits of the protein kinase-like fold.

The few published studies and our current work on the first five Nek kinases suggest that several signaling proteins have distinct associations with the PFR, the different flagellar axonemes, or the unique ventral disk and median bodies. The latter, like the basal bodies and flagella, are microtubule-based. Several signaling proteins are shared between the caudal flagella with its associated structures and the disk - Neks 16279 and 92498, ERK1 and PP2Ac (protein phosphatase 2A) - suggesting that they may function in the same signaling pathway.

Understanding the replication and segregation of the two nuclei and complex cytoskeleton during the *Giardia *cell cycle and life cycle has been challenging [67]. The flagellar basal bodies migrate laterally during mitosis to become spindle poles. Several of the *Giardia *kinases and phosphatases studied to date localize to the basal bodies during interphase, but most have not yet been studied in mitosis or differentiation (Table 1, Figure 1) and only AurK, PKA and PP2A phosphatase have been partially functionally analyzed. The strong pSer and pThr staining within the flagellar axonemes suggests that substrates may be phosphorylated in the basal bodies before incorporation into the axonemes. Analyses of flagellar-associated kinases and signaling may help better understand the roles of the four flagellar pairs in *Giardia *swimming, attachment, and detachment, which are central to disease [68], as well as to better understand the roles of this almost universal organelle.

Taken together, our data may help to prioritize future functional kinase studies, elucidate the signaling underlying the cell and life cycles and provide new drug targets to treat *Giardia *infections. Protein kinases are proven drug targets, and the high divergence of *Giardia *sequences suggests that specific inhibitors could be developed that have minimal activity against human kinases. Our findings help define the minimal kinase complement of a single-celled eukaryote with a complex life and cell cycle and add to our understanding of *Giardia *biology, pathogenesis, and evolution.

## Materials and methods

### Software, data sets and databases

The *G lamblia *genome assemblies for all three strains were from release 2.3 of GiardiaDB [69]. Sequenced strains are from ATCC, accession numbers 50803 (assemblage A, WB clone C6), 50581 (assemblage B, clone GS) and GLP15 (assemblage E, clone p15). *T. vaginalis *sequences were from release 1.2 of TrichDB [70], and *L. major *from release 2.5 of TriTrypDB [71].

### Sequence analysis

We constructed profile HMMs for the ePK, PIKK, RIO, ABC1, PDK, and alpha-kinase families with HMMer and used these to search the ORF, genomic, and EST sequences using Decypher hardware-accelerated HMMer implementation from Time Logic (Carlsbad, CA, USA). Divergent Neks were identified with several Nek-specific HMMs and Blast searches, followed by manual inspection for conserved kinase motifs. Final predicted kinase sequences were searched against the Pfam HMM profiles, using both local and glocal models. All matches with P scores < 0.01 were accepted and all matches with scores of 0.01 to 1.0 were evaluated in comparison with known, homologous sequences, inspection of the domain alignment, and reference to the literature. *L. major *sequences were classified in part using psi-blast with orthologous sequences from other kinetoplastids, and *T. vaginalis *expansions were also classified using psi-blast profiles built from paralogs. Signal peptides were detected using SignalP and transmembrane regions using TM-HMM [72] and coiled-coil domains according to Lupas *et al*. [73]. Nek kinase domains were aligned with ClustalW [74] and HMMalign [75], and then extensively edited by hand using JalView [76]. The Nek tree was built using the ClustalW neighbor-joining algorithm and colored by hand using Adobe Illustrator.

### Cultivation of *Giardia*

*G. lamblia *trophozoites (strain WB, clone C6, ATCC 50803) were cultured in modified TYI-S-33 medium with bovine bile [77,78].

### Western blot

Cells were washed with ice cold PBS and cell proteins were precipitated in 6% TCA (trichloroacetic acid) for 2 hours on ice. Protein pellets were resuspended in reducing SDS-PAGE sample buffer, neutralized with NaOH, boiled for 5 minutes and stored at -80°C until use. Protein concentrations were determined by the Bradford method (Biorad, Hercules, CA, USA). Proteins were separated by 4-20% SDS PAGE and transferred to PVDF filters. Filters were blocked with 1% milk in PBS supplemented with 0.1% Tween 20 (PBS-Tween) and incubated for 1 hour with the FITC-labeled mouse monoclonal antibodies against pSer, pThr or pTyr (Sigma, St. Louis, MO, USA) in 1% milk. Blots were then washed four times with PBS-Tween and incubated with secondary antibody (goat anti-mouse-horse radish peroxidase (HRP)) for 1 hour. The signal was developed with ECL-plus (GE Healthcare, Waukesha, WI, USA). As a protein loading control, blots were reprobed with the mouse monoclonal anti-taglin antibody [79] and goat anti-mouse-HRP. As a control for antibody specificity, antibodies were incubated with pSer, pThr or pTyr conjugated to bovine serum albumin (Sigma), respectively, prior to immunolabeling of filters. As an additional control, total *Giardia *lysates were dephosphorylated with protein phosphatase λ (New England Biolabs, Ipswich, MA, USA) according to the manufacturer's protocol. Both controls eliminated signal on western blot, confirming specificity of the antibodies (data not shown).

### Epitope tagging of proteins

The region containing the promoter (> 100 base pairs upstream of the start codon) and coding sequences for Orf_5375 were amplified from *G. lamblia *strain WB clone C6 (ATCC 50803) genomic DNA with the primers 5'-taagggccccagcatctagctgaatgccga-3' and 5'-taagatatccatcttatacttgtaagcgcc-3', Orf_92498 with primers 5'-gggcccccggatgcgcgtctgttg-3' and 5'-gatatccctgacagtattgaacctgtcc-3', Orf_16279 with primers 5'-gggcccggatccgaggtcatgcgc-3' and 5'-gatatcagaaaggcgtctctgcgtcaaaac-3', Orf_101534 with primers 5'-gggcccggcctgactgcgcatgc-3' and 5'-gatatcctgtctgagcatctcgcacagc-3', and Orf_15409 with primers 5'-tttaagcttcccctgccgctgagtgaacat-3' and 5'-tttgggccccaggttcaggacctcacgcac-3'. The PCR products and the vector encoding the carboxy-terminal AU1 tag (Orf_15409) [80] or HA tag (all other Neks) [81] were digested with the respective restriction enzymes. Digested inserts and vectors were gel extracted using a QIAquick Gel Extraction Kit (Qiagen, Venlo, The Netherlands), and ligated overnight at 14°C. Plasmids were transformed into *Escherichia coli *JM109 (Promega, Fitchburg, WI, USA}). Bacteria were grown overnight in Luria broth and plasmid DNA was purified using a Maxiprep kit (Qiagen) and sequenced (Etonbio, San Diego, CA, USA}). Trophozoites were electroporated with 50 μg plasmid DNA and transfectants were maintained through puromycin selection [82]. Base pairs 1.051 to 1,158 from the ankyrin repeat region of Orf_15409 were deleted by linking the upstream and downstream PCR products together with the internal primers 5'-agtccacatgtactggtctgtggaccctgcctggtg-3' and 5'-tccacagaccagtacatgtggactgcaaccatgtat-3'.

### Immunofluorescence analysis

Trophozoites were harvested by chilling and allowed to adhere to coverslips at 37°C for 10 minutes. Whole trophozoites were fixed *in situ *with methanol (-20°C), permeabilized for 10 minutes with 0.5% Triton X-100 in PBS [13] and blocked for 1 hour in 5% goat serum, 1% glycerol, 0.1% bovine serum albumin, 0.1% fish gelatin and 0.04% sodium azide. Coverslips were subsequently incubated for 1 hour with the FITC-labeled mouse monoclonal antibodies against pSer, pThr or pTyr (Sigma) or with the rat anti-HA-FITC (Roche, Indianapolis, IN, USA). Cells that were expressing AU1-tagged Nek (Orf_15409) were incubated with the primary antibody mouse anti-AU1 for 1 hour, washed four times over 20 minutes, and incubated with the goat anti-mouse Alexa 488 secondary antibody (Invitrogen, Carlsbad, CA, USA). Coverslips were washed, postfixed with 4% paraformaldehyde, rinsed with PBS and mounted with Prolong Gold with DAPI (Molecular Probes, Eugene, OR, USA). As a control for antibody specificity, antibodies were incubated with pSer-, pThr- or pTyr-labeled albumin (Sigma), respectively, prior to immunolabeling. Staining was monitored and photographed on a Nikon Eclipse E800 microscope with an X-Cite™ 120 fluorescence lamp and 1,000 × magnification (Nikon Instruments Inc.). Confocal images were taken with the Leica TCS SP5 system attached to a DMI 6000 inverted microscope (Leica).

## Abbreviations

CDK: cyclin-dependent kinase; DAPI: 4',6-diamidino-2-phenylindole; DNAPK: DNA protein kinase; EST: expressed sequence tag; FITC: fluorescein isothiocyanate; GSK: glycogen synthase kinase; HA: hemagglutinin; HCMP: high cysteine membrane protein; HMM: hidden Markov model; MAPK: mitogen-activated protein kinase; ORF: open reading frame; PBS: phosphate-buffered saline; PFR: paraflagellar rods; PIK: phosphatidyl inositol kinase; PIKK: phosphatidyl inositol 3' kinase-related kinase; PK: protein kinase; pSer: phosphoserine; pThr: phosphothreonine; pTyr: phosphotyrosine; SAGE: serial analysis of gene expression; SP: Ser-Pro; TA: TALM-ankyrin; TK: tyrosine kinase; TKL: tyrosine kinase-like; TOR: target of rapamycin; VSP: variant-specific surface protein.

## Authors' contributions

FDG, GM, DSR, and SS conceived of the study, including its design and coordination. GM and FDG wrote the manuscript with contributions from TL, AS, and MD. DSR cataloged the initial WB kinome, and MD, GM, and YZ carried out extensive curation and computational and phylogenetic analysis of all five kinomes. TL performed the immunoblot identification and immunolocalization of phosphorylated proteins (Figures 3 and 4). TL and AS carried out the molecular genetic and cell biologic studies of the Nek kinases and prepared Figures 1 and 3 to 5 and Table 1. All authors contributed to the editing of the manuscript.

## Supplementary Material

Additional file 1 **Table S1**. Detailed annotation of all kinases in all three strains, including sequences, SAGE expression, classification, and catalytic ability. Click here for file

Additional file 2 **Text S1**. Supplemental methods and notes. Click here for file

Additional file 3 **Table S2**. Draft kinomes of *Trichomonas vaginalis *and *Leishmania major*. Click here for file

Additional file 4 **Table S3**. Definition of domain names and abbreviations. Click here for file

Additional file 5 **Figure S4**. Nek kinase tree, colored and annotated. Click here for file

Additional file 6 **Alignment S1**. Nek kinase domain alignment. Click here for file

Additional file 7 **Figure S1**. Logo alignment comparing patterns of conserved residues in *Giardia *and non-*Giardia *Neks. Click here for file

Additional file 8 **Figure S2**. Tree of Nek kinases showing gains and losses between strains. Click here for file

Additional file 9 **Figure S3**. Logo alignment comparing patterns of conserved residues in *Giardia *TALM-ankyrin repeats and human ankyrin repeats. Click here for file
