# Supplementary material for: The minimal kinome of Giardia lamblia illuminates early kinase evolution and unique parasite biology
Source: Genome Biol. 2011 Jul 25;12(7):R66. doi: 10.1186/gb-2011-12-7-r66 (PMC3218828; doi:10.1186/gb-2011-12-7-r66)
Supplement: Additional file 2 — Text S1. Supplemental methods and notes. [file gb-2011-12-7-r66-S2.DOC]

## Supplemental Methods and Notes for Manning et al, Giardia Kinome

**Prediction and Classification of Protein Kinases**

We used HMMs and homology to known kinases in other organisms to predict putative kinases in the gene predictions and raw genomic sequence downloaded from GiardiaDB release 2.3. After the initial identification of kinase loci, extensive manual analysis was undertaken to annotate and curate all kinases in strain WB, including identification of pseudogenes by extending homology in genomic sequences. Strains GS and P15 were annotated using WB and were not probed for pseudogenes. The GS assembly in particular had many fragmentary genes due to short contigs, so even incomplete fragments were annotated as genes. 265 of the 314 sequences reported in Table S1 (protein and non-protein kinases and pseudogenes) are identical to GiardaDB release 2.3. For 14 sequences, there was no GiardiaDB prediction and for the other 35, we extended a short annotated ORF from GiardiaDB to a longer region of homology. In 17 cases, the longer sequences overlap multiple ORF predictions from GiardiaDB. 28 of the extensions include either frameshifts or stops and are designated as pseudogenes. Strains P15 and GS have 18 and 15 possible pseudogenes respectively, although we did not attempt to analyze or extend the pseudogenes in either strain.

We attempted to classify orthologous sequences between the three strains. The majority of sequences, including the entire ‘core’ (non-Nek) kinome, are obvious three-way orthologs, with representatives in all three strains. These have been annotated as ‘1:1:1’ in the assemblage orthology column of Table S1. Of the remaining genes, seventeen are in only strains WB and P15 (which are closer to each other than to strain GS), and have been annotated as ‘AE’ in the assemblage orthology column (for assemblages ‘A’ and ‘E’ – strains WB and P15). An additional seven genes are present in only WB and GS, and thus annotated as AB (assemblage B is strain GS), and four genes in only GS and P15, and thus annotated BE. Finally there are 42 genes in complex clusters annotated as ‘C’.

In strain WB we found 5 100% identical loci. One of these appears to be a legitimate duplication, and is not 100% identical at the nucleotide level - a single base has synonymously changed - and only the ORF has been duplicated, with no sequence similarity in the flanking genomic region. This locus has also been duplicated before, there is another ORF that is >99% identical. These three ORFs are members of the expanding NEK GL4 subfamily. The remaining 4 duplicate loci are more complex and all have some assembly issue associated with them that makes it difficult to be certain of their validity. One of the duplicates is 100% identical, but the identical region extends far past the bounds of the ORF; in another, only the ORF appears to have been duplicated, but the duplicate sequence has been split across two contigs and is missing a small region of sequence. In the remaining two, the duplicate region is a subset of the original ORF, making the duplicate a fragment. In one of these the 100% identical region extends upstream past the N-term of the ORF, but terminates in the middle of the ORF. Such assembly artifacts are relatively rare for strains WB and P15, and only slightly more common in GS (where there as 6 such cases) as supported by the perfect 1:1:1 orthology in the core kinome.

We searched the kinome for coiled-coil regions using paircoil (<http://groups.csail.mit.edu/cb/paircoil2/>), using a window size of 28 and a coil e-value cutoff of 0.01. No potential coil region less than 5 amino acids long was considered. We found a total of 41 kinases in strain WB with coiled-coil regions (between 1 and 3 distinct coiled-coil regions in each sequence), all but two of which were Neks (TableS1, ‘# Coils’ column). The two non Neks with coil hits are the *Giardia*-specific Other-Unique kinase, Orf_14392, and Fray (Orf_10609).

**Divergence Rate of *Giardia* Kinases**

In order to quantify the sequence divergence of Giardia vs other major eukaryotic groups we compared the kinase domains of 9 orthologous kinases (PDK1, CDC2, CK2, Erk1, SRPK, Aur, Bud32, GCN2, and YSK) from 8 species (*Amphimedon.queenslandica*, *Giardia*, *Monosoga brevicollis*, *Oryza sativa*, *Saccharomyces cerevisiae*, *Selaginella moellendorffii*, *Tetrahymena thermophila*, and *Trichomonas vaginalis*) by BlastP. We averaged the HSP identity over the length of the target (the Human ortholog kinase domain). We found that *Giardia* has an average kinase domain sequence identity to Human of just 40%, compared with 46% in *Trichomonas* and *Tetrahymena*, and between 49% and 50% in *Oryza*, *Selaginella*, and *S.cerevisiae*. *A. queenslandica* scored the highest with 62% identity.

**Notes on Figure 2**

Two outgroups (two of chromalveolates/plants/unikonts) are required to designate a kinase as primordial, to minimize effects of possible horizontal transfer, convergent evolution, or annotation error. For that reason, the tree omits LKB (found in unikonts and with tentative homologs in *T. vaginalis* but not in other lineages), and CIPK and CK1y (found in plants and excavates). This classification does not include the very weak ATM/ATR-like gene from *Giardia*.
