# Supplementary figures and images for: The minimal kinome of Giardia lamblia illuminates early kinase evolution and unique parasite biology
Source: Genome Biol. 2011 Jul 25;12(7):R66. doi: 10.1186/gb-2011-12-7-r66 (PMC3218828; doi:10.1186/gb-2011-12-7-r66)

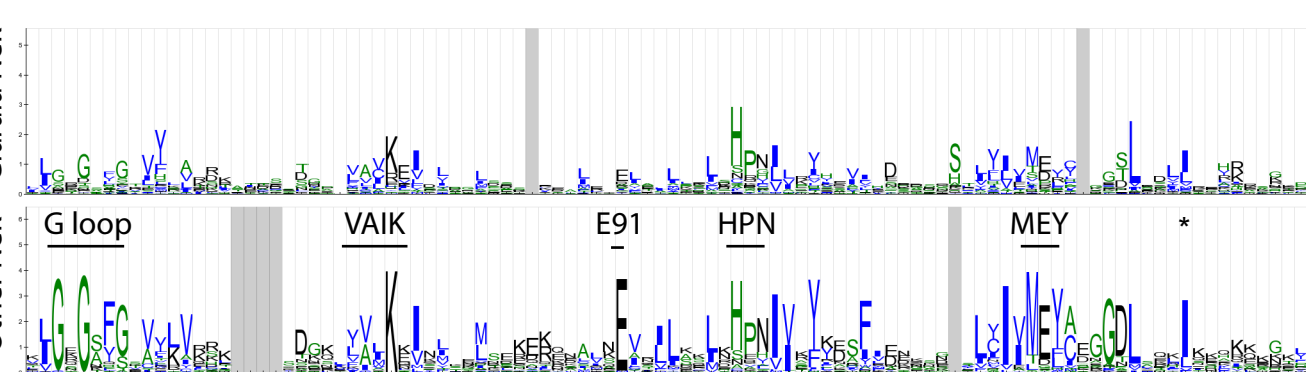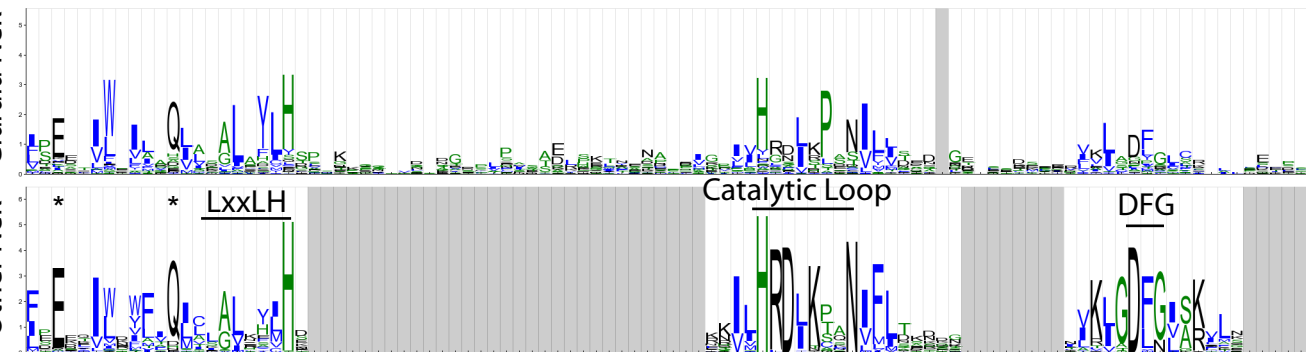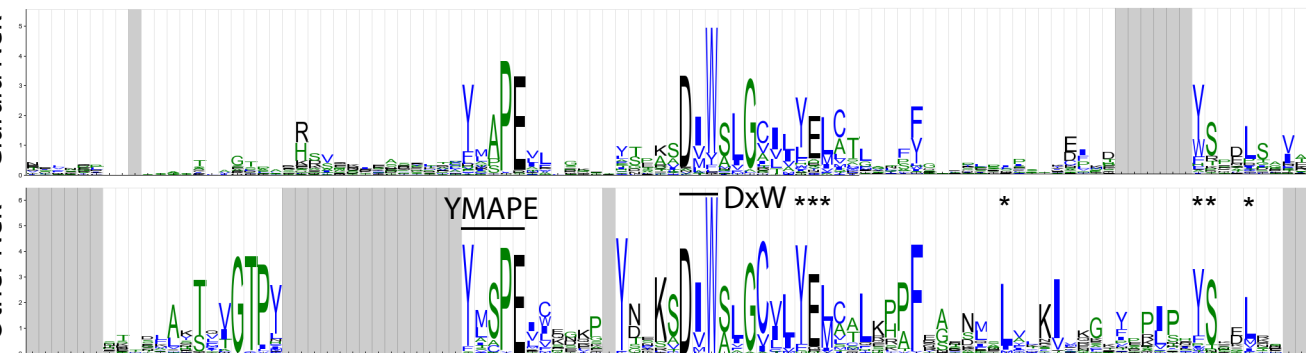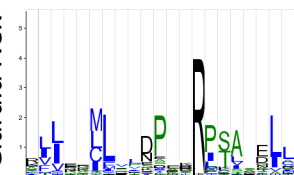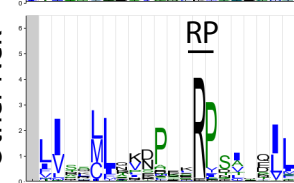

Supplement: Additional file 7 — Figure S1. Logo alignment comparing patterns of conserved residues in Giardia and non-Giardia Neks. [file gb-2011-12-7-r66-S7.PDF]

A. All Neks

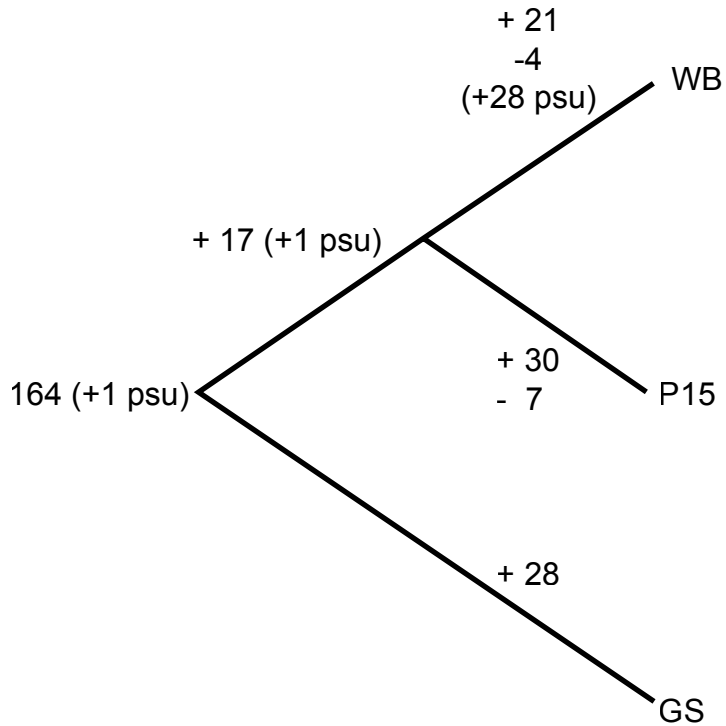

A. Nek-GL4

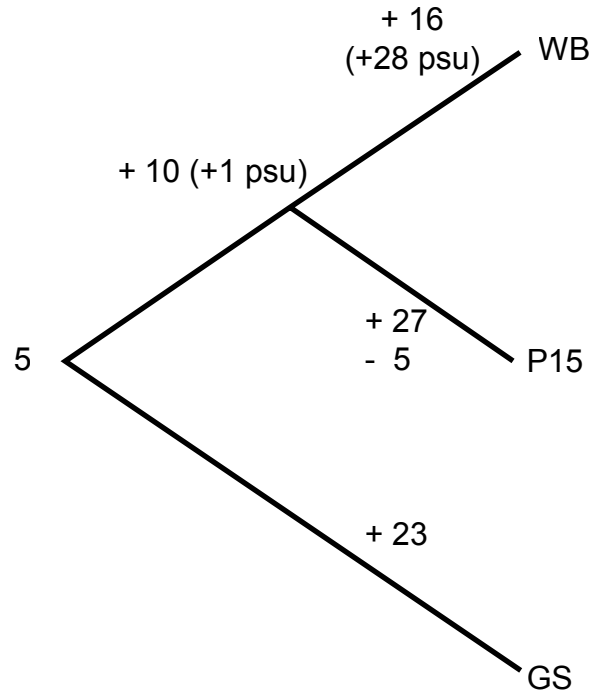

Supplement: Additional file 8 — Figure S2. Tree of Nek kinases showing gains and losses between strains. [file gb-2011-12-7-r66-S8.PDF]

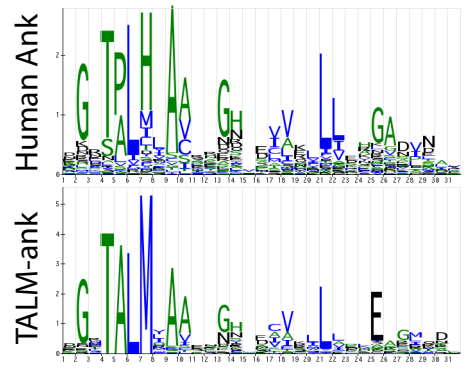

Supplement: Additional file 9 — Figure S3. Logo alignment comparing patterns of conserved residues in Giardia TALM-ankyrin repeats and human ankyrin repeats. [file gb-2011-12-7-r66-S9.PDF]
