# Supplementary material for: The minimal kinome of Giardia lamblia illuminates early kinase evolution and unique parasite biology
Source: Genome Biol. 2011 Jul 25;12(7):R66. doi: 10.1186/gb-2011-12-7-r66 (PMC3218828; doi:10.1186/gb-2011-12-7-r66)
Supplement: Additional file 5 — Figure S4. Nek kinase tree, colored and annotated. [file gb-2011-12-7-r66-S5.PDF]

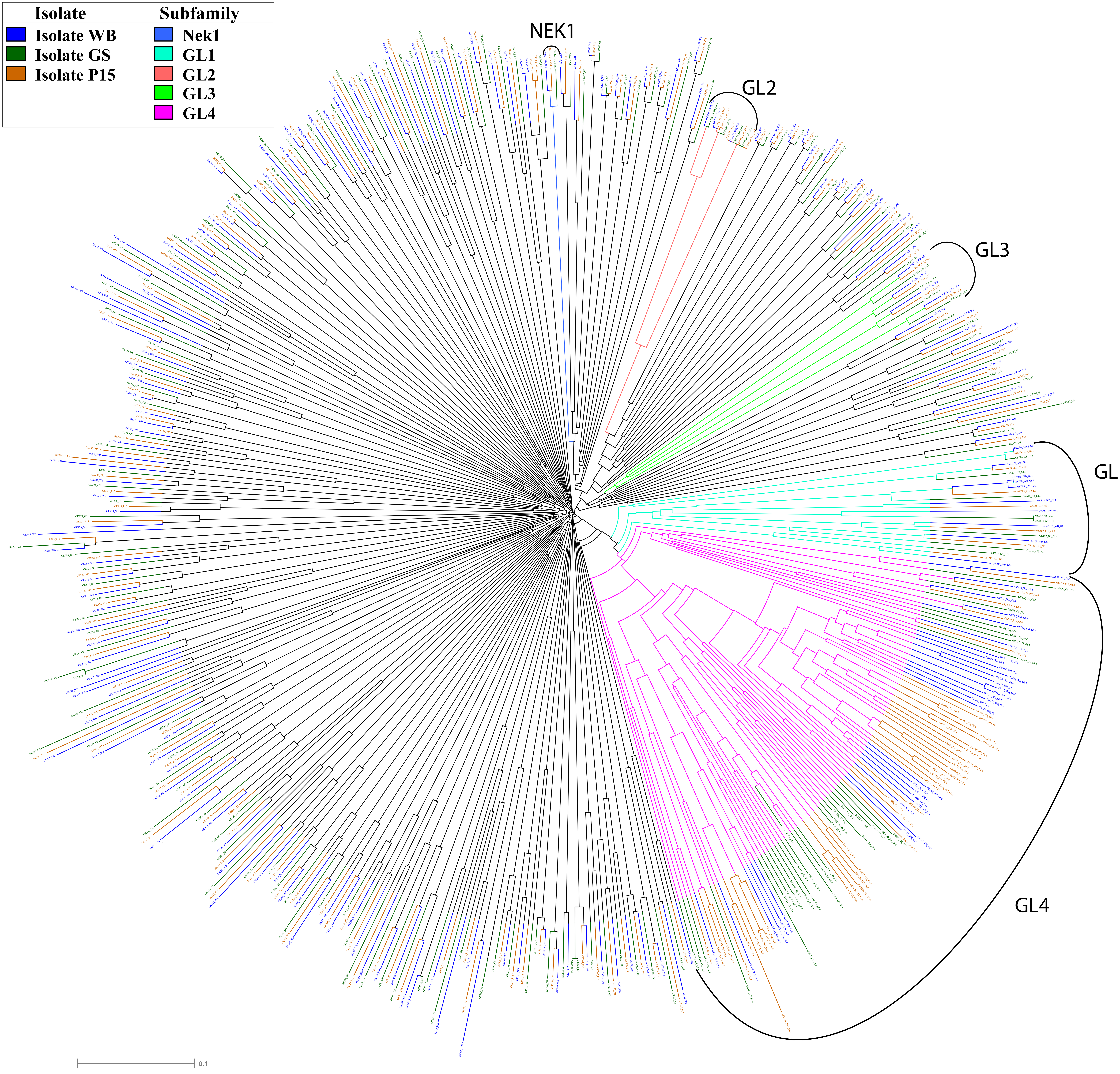

Isolate
Isolate WB
Isolate GS
Isolate P15
Subfamily
Nek1
GL1
GL2
GL3
GL4
NEK1
GL2
GL3
GL1
GL4
0.1
